# Serum Proteomic and Metabolomic Signatures of High Versus Low Physical Function in Octogenarians

**DOI:** 10.1111/acel.70002

**Published:** 2025-03-10

**Authors:** Ceereena Ubaida‐Mohien, Ruin Moaddel, Sally Spendiff, Norah J. MacMillan, Marie‐Eve Filion, Jose A. Morais, Julián Candia, Liam F. Fitzgerald, Tanja Taivassalo, Paul M. Coen, Luigi Ferrucci, Russell T. Hepple

**Affiliations:** ^1^ Intramural Research Program, National Institute on Aging National Institutes of Health Baltimore Maryland USA; ^2^ Children's Hospital of Eastern Ontario Research Institute Ottawa Canada; ^3^ Research Institute of the McGill University Health Centre McGill University Montreal Canada; ^4^ Department of Physical Therapy University of Florida Gainesville Florida USA; ^5^ Department of Physiology and Aging University of Florida Gainesville Florida USA; ^6^ Translational Research Institute Advent Health Orlando Florida USA

**Keywords:** aging, high functioning, inflammation, integrated stress response, kynurenine, low functioning, master athletes, mitochondria, SASP, serum proteomics

## Abstract

Physical function declines with aging, yet there is considerable heterogeneity, with some individuals declining very slowly while others experience accelerated functional decline. To gain insight into mechanisms promoting high physical function with aging, we performed proteomics, targeted metabolomics, and targeted kynurenine‐focused metabolomic analyses on serum specimens from three groups of octogenarians: High‐functioning master athletes (HF, *n* = 16), healthy normal‐functioning non‐athletes (NF, *n* = 12), and lower functioning non‐athletes (LF, *n* = 11). Higher performance status was associated with evidence consistent with: Lower levels of circulating proinflammatory markers, as well as unperturbed tryptophan metabolism, with the normal function of the kynurenic pathway; higher circulating levels of lysophosphatidylcholines that have been previously associated with better mitochondrial oxidative capacity; lower activity of the integrated stress response; lower levels of circulating SASP protein members; and lower levels of proteins that reflect neurodegeneration/denervation. Extending the observations of previous studies focused on the biomarkers of aging that predict poor function, our findings show that many of the same biomarkers associated with poor function exhibit attenuated changes in those who maintain a high function. Because of the cross‐sectional nature of this study, results should be interpreted with caution, and bidirectional causality, where physical activity behavior is both a cause and outcome of differences in the biomarker changes, remains a possible interpretation.

## Graphical abstract

Circulating factors are strongly implicated in modulating biological aging. In this study we show that the serum proteomic and metabolomic profile of high functioning octogenarians suggests lower activation of SASP, preservation of the tryptophan‐kynurenic pathway, higher lipophosphatidyl cholines promoting mitochondrial function, and reduced neurodegeneration and denervation. Figure created in Biorender. Hepple, R. (2025).

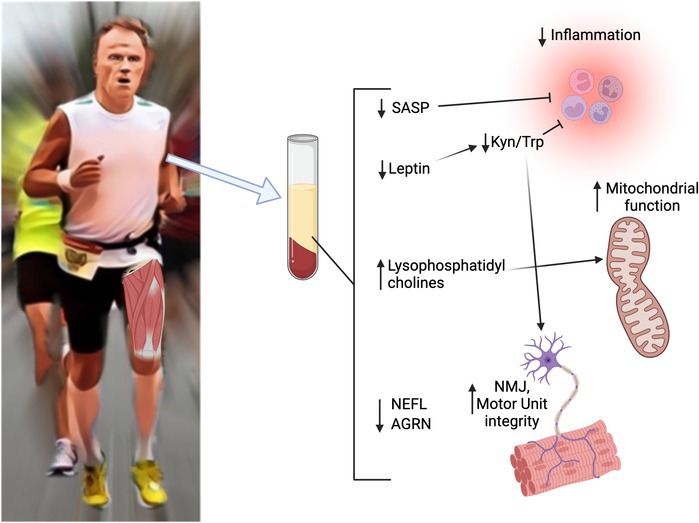

## 1 Introduction

Octogenarian master athletes are the living proof that preserving a high level of physical function in advanced age is possible. Maintaining a high level of physical function later in life is likely influenced by a coalescence of genetic, environmental, and behavioral factors, in particular concerning the nervous, musculoskeletal, and metabolic systems. The study of circulating biomarkers in older individuals with contrasting levels of physical function may offer insights into the potential mechanisms that counteract the accumulation of molecular and cellular damage associated with aging and are permissive of a healthy lifespan. Consistent with this suggestion, preclinical models of heterochronic parabiosis experiments find that circulating factors play pivotal roles in determining organismal aging and metabolic health (Katsimpardi et al. 2014; Pamplona et al. 2023; Rebo et al. 2016).

To gain insights into potential human‐relevant mechanisms for preserving physical functions with aging, we performed proteomics and targeted metabolomic analyses on blood serum specimens collected from high‐functioning octogenarian master athletes (HF) in comparison to serum from healthy normal‐functioning octogenarian non‐athletes (NF) and low‐functioning octogenarian non‐athletes (LF). Based on recent literature suggesting that the kynurenine synthetic pathway contributes to functional decline with aging (Hetherington‐Rauth et al. 2024; Jang et al. 2020; Westbrook et al. 2020), we also performed a targeted kynurenine‐focused metabolomic analysis. Using these data, we explored the proteomic, metabolomic, and kynurenine profiles in these functionally distinct groups. In addition, we compared this serum proteome with the plasma aging proteome (Tanaka et al. 2018) and from the same HF and NF participants the skeletal muscle proteome (Ubaida‐Mohien et al. 2022). The aim of this comprehensive, multiomic analysis was to gain insights to the potential physiological mechanisms involved in preserving systemic health and physical function in advanced age that could be explored in more detail in large cohort studies in the future. The pathways involved in these physiological mechanisms may become targets for preventive intervention aimed at maximizing physical function in old age.

## 2 Results

### 2.1 Preserving Physical Function in Advanced Age: Comparison of Serum Proteins and Metabolites Among HF, NF, and LF Participants

**FIGURE 1:**
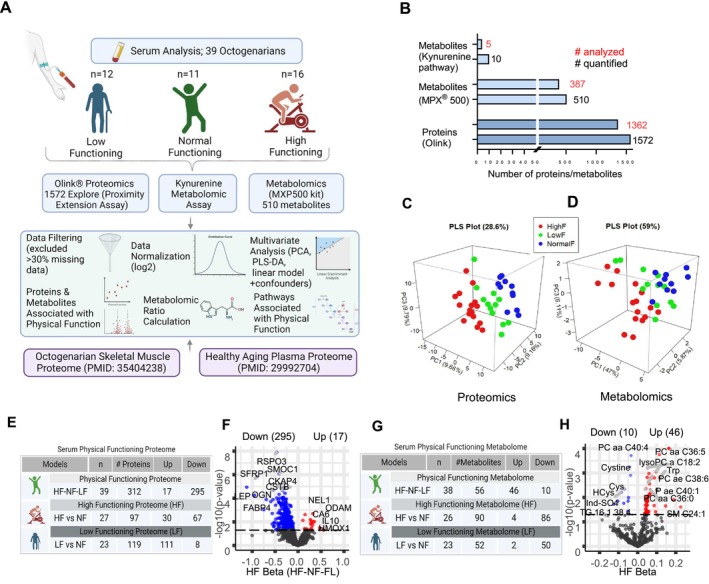
Comparison of serum proteins and metabolites among HF, NF, and LF participants. (A) Serum study design and workflow. Serum samples from 39 octogenarians were used to perform proteomics, kynurenine‐targeted metabolomics, and metabolomic analysis. Skeletal muscle data published previously from the same HF and NF participants were used for corroborative analysis. To validate physical function in relation to aging effect in circulation, plasma proteome data published previously were used. (B) Number of proteins and metabolites were quantified and analyzed. (C) Principal least‐square analysis of the physical function groups shows a separation among groups by proteins and (D) metabolites. 1362 proteins and 387 metabolites were used for the PLS analysis. (E) Three linear model analyses adjusted for age and sex were performed. Representative table of PF, HF, and LF serum proteomes. Number of significant proteins (*p* < 0.05) identified from each linear model is shown. (F) PF serum proteins, which are lower (left) and higher (right), are shown, *p* < 0.05 proteins represented in red (up)/blue (down) circles. The horizontal line marks the significance cutoff at *p* = 0.05. The top significant proteins are labeled. (G) Representative table of PF, HF, and LF serum metabolomes. Number of significant metabolites (*p* < 0.05) identified from each linear model is shown. (H) PF serum metabolites, which are under‐ (left) and over (right)‐represented are shown, *p* < 0.05 represented in red/blue circles. The horizontal line marks the significance cutoff at *p* = 0.05. Top significant metabolites are labeled.

To investigate the influence of physical function on circulating proteins and metabolites, we obtained serum samples from three groups of octogenarians: 16 HF master athletes, 11 NF non‐athletes, and 12 LF non‐athletes. Briefly, HF individuals were competitive master athletes in track and field (*n* = 15) or cycling (*n* = 1) with a mean walking speed of 1.60 ± 0.22 m⋅s^−1^; NF individuals were non‐athletes with a mean walking speed of 1.25 ± 0.14 m⋅s^−1^; and LF individuals were non‐athletes with a mean walking speed of 0.83 ± 0.09 m⋅s^−1^. There was no significant difference in the age or sex distribution among groups, and their summary characteristics are presented in Table 1. Individual subjects' walking speed, grip strength, and VO_2peak_ are shown in Figure S1. As expected, the HF had a significantly higher VO_2peak_ compared to NF and LF. Study participants were septuagenarians, octogenarians, and nonagenarians, with the age distribution of 75–93, 75–87, and 71–91 years, for high‐, normal‐, and low‐functioning, respectively. However, considering the average age of participants, and to simplify the narrative, we use the term “octogenarians” to refer to all participants in this study. An overview of the serum analyses is presented in Figure 1A.

**TABLE 1:** Characteristics of HF, NF, and LF octogenarians. BMI, Body Mass Index; Group differences were assessed by Chi‐squared test for categorical variables and ANOVA for continuous variables; age, BMI, and walking speed, grip strength, and VO_2peak_ data were presented as averages of the group. *p < 0.05 versus other groups.

Physical function group	High‐functioning	Normal‐functioning	Low‐functioning	*p*
*n*	16	11	12	—
Age (years)	80.2 ± 4.7	80.5 ± 3.3	78.5 ± 7.2	0.38
Sex (male)	50%	63.6%	41.6%	0.85
BMI	22.3 ± 2.5*	26.9 ± 3.3	28.3 ± 5.6	0.007
Sprint/endurance	8/8	NA	NA	—
Walking speed (m/s)	1.6 ± 0.22*	1.25 ± 0.14*	0.83 ± 0.09*	< 0.0001
Grip strength, kg	29.1 ± 10.5	32.7 ± 20.3	23.1 ± 10.5	—
VO_2peak_, mL/min/kg	34.4 ± 8.7*	20.2 ± 3.0	16.5 ± 3.3	< 0.0001

After excluding proteins and metabolites that fell below the limit of detection (LOD), we analyzed 1362 proteins, 387 metabolites, and 5 targeted metabolites collected from a total of 39 participants (Figure 1B). To explore group differences in protein/metabolite abundances, all proteins and metabolites were clustered into physical functioning groups using partial least‐squares (PLS) analysis, as depicted in Figure 1C,D. The spread of proteomic data among the HF and LF groups highlights the heterogeneity within these groups. Notably, the metabolomic data showed a higher variance among the HF and LF participant groups, possibly because metabolites undergo short‐term changes that contribute to increased variability. Linear model analyses adjusted for age and sex were employed to characterize proteins associated with higher functioning (the continuum from HF to NF to LF), proteins associated with high compared to normal functioning (HF vs. NF), and proteins associated with low compared to normal functioning (LF vs. NF) (Figure 1E). A similar analytical pipeline was used to compare serum metabolites (Figure 1G). The results from these analyses are referred to as *physical functioning* (PF) proteome/metabolome (HF to NF to LF), *high‐functioning* (HF) proteome/metabolome (HF vs. NF), and *low‐functioning* (LF) proteome/metabolome (LF vs. NF). For the PF proteome/metabolome, three groups were simultaneously compared, whereas for the HF and LF proteomes/metabolomes, two respective groups were compared.

Among the total 1362 measured proteins, 312 were significantly different among the HF, NF, and LF groups, collectively referred to as the PF proteome (Figure 1E,F, Table S1). Notably, a group of 97 proteins was significantly different between HF vs. NF, referred to as the HF proteome (Figures 1E and S2B, Table S1), and 119 proteins were significantly different between LF vs. NF, referred to as the LF proteome (Figure 1E, Table S1). Of note, 38% of the differentially expressed proteins across the three analyses was shared among the PF and HF/LF groups (Figure S2A). The statistical significance of protein abundance among groups (HF beta and *p*‐value < 0.05) reported here was derived from PF proteome analysis (HF, NF, and LF groups) unless otherwise reported. The top significant proteins RSPO3 (HF beta = −0.46, *p* = 1.3E‐8) and SMOC1 (HF beta = −0.41, *p* = 8.38E‐07) were lower in HF compared to NF and LF. RSPO3 is an important determinant of the peripheral adipose tissue storage capacity (Garritson and Boudina 2021; Loh et al. 2020), while SMOC1 appears to be consistently identified as a key protein in Alzheimer's disease, as multiple proteomic studies have found higher SMOC1 in brain tissue and CSF, as well as associations with amyloid pathology (Roberts et al. 2023).

Of the 387 quantified metabolites, 56 were differentially represented metabolites in the PF metabolome (10 lower and 46 higher with a higher physical function), 90 in the HF metabolome (86 lower and 4 higher with a higher physical function), and 52 metabolites in the LF metabolome (50 lower and 2 higher with a lower physical function) (Figure 1G, Table S2). Notably, phosphatidylcholines (PCs), lysophosphatidylcholines (LysoPCs), and tryptophan (Trp) were the most significant metabolites with a higher abundance in HF (HF beta = 0.05, *p* = 0.0006), while cystine, cysteine (Cys), and homocysteine (HCys) were the most significant metabolites with a lower abundance in the HF group (Figure 1H).

### 2.2 High Physical Functioning in Octogenarians Is Associated With Under‐Representation of the Major Innate and Adaptive Immunity Pathways With Signs of Preserved Immune Response

**FIGURE 2:**
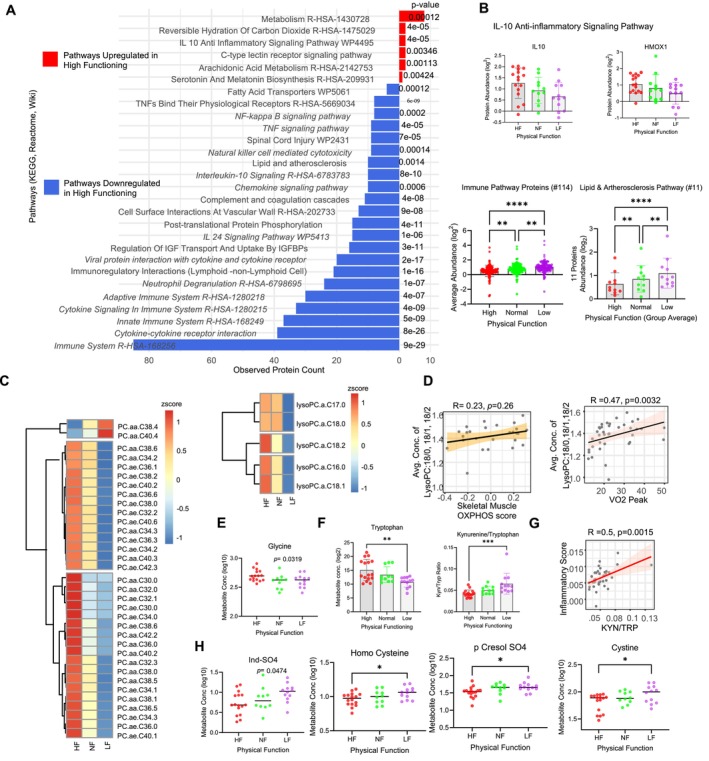
Proteins and metabolites associated with major innate and adaptive immunity pathways and immune response. (A) Pathway enrichment analysis (KEGG, Rectome, and Wiki pathways) of PF proteome shows top significant pathways that were lower (blue) and higher (red) in HF participants. The number of PF proteins representing the pathway is shown in *x*‐axis, and the *p*‐value for each pathway is in the *z*‐axis. Italicized pathway names are immune system pathways used to create “inflammatory score”. (B) Protein abundance of IL‐10 anti‐inflammatory signaling pathway proteins are shown; each circle represents a participant (*n* = 39), and the significance of the analysis is calculated by ANOVA (Kruskal–Wallis test) (top). “Inflammatory score” proteins and lipid and atherosclerosis pathway proteins (*n* = 11) are averaged and shown for each participant. (C) PF serum metabolites show an association with higher level of several PC's and LPC's in PF group corresponding with decreased proteomic representation of proinflammatory pathways. (D, left) Serum metabolite LysoPC correlation with the skeletal muscle OXPHOS score. Average concentration of Lyso PC18:0/1/2 were correlated with the OXPHOS score. (Right). Lyso PC18:0/1/2 versus VO_2peak_. Spearman correlation and significance of the correlation are shown on top. (E) Log2 concentration of Glycine with physical function. (F) Log2 concentration of Tryptophan with physical function. Ratio of Kyn/Trp metabolite. Significance of the metabolites was shown as (*p < 0.05) measured from ANOVA analysis. (G) Correlation of Kyn/Trp with inflammatory score derived from the 114 immune pathway proteins from Figure 2A. (H) Individual metabolites involved in the interface of inflammatory/immune response are shown. The *p*‐value significance of the metabolites is shown as (*p < 0.05) measured from ANOVA analysis.

In the PF proteome, few enriched pathways were found to be over‐represented in HF compared to NF and LF groups (Figure 2A, red). “Metabolism” was the most significant pathway over‐represented in HF participants, represented by proteins such as PON3, DDC, HPGDS, APOM, GLB1, HMOX1, CA6, and CA14. PON3 is an antioxidant enzyme shown to be higher during exercise training in rats (Romani et al. 2009), while DDC is an enzyme required for dopamine synthesis. Interestingly, the IL‐10 anti‐inflammatory signaling pathway, led by proteins IL‐10 and HMOX1 (heme oxygenase‐1), which serves as a downstream target of the transcription factor HIF‐1α (hypoxia‐inducible factor‐1α)—a key regulator of the body's response to hypoxia (Dunn et al. 2021)—was notably higher in HF (Figure 2B, top). Additionally, pathways associated with inflammation, with proinflammatory cytokines, chemokines, and interleukins as leading proteins, were lower in HF compared to NF and LF (Figure 2A, blue). To better understand this finding, we averaged the level of 114 immune system proteins (Figure 2A; selected pathways are italicized; see the Methods section for details) and plotted average values across HF, NF, and LF (Table S3). The resulting “inflammatory score” was significantly lower in HF compared to the other two groups (Figure 2B, bottom left). These results suggest that high physical function in advanced age is associated with a protective effect against the biological mechanisms that unleash a proinflammatory state during the aging process. In contrast, the higher “inflammatory score” in NF and LF groups compared to HF suggests activation of the innate and adaptive immune system associated with lower physical function (Figure 2B, bottom left), likely as a chronic response to accumulating molecular damage (Singh et al. 2023). The lipid and atherosclerosis pathway led by 11 proteins was lower in HF. The average of these 11 proteins is shown in Figure 2B (bottom right), suggesting that HF is associated with a lower burden of metabolic traits associated with the risk of cardiovascular diseases and their complications.

Enrichment analysis identified two IL‐10 pathways: IL‐10 anti‐inflammatory signaling pathway (from WikiPathways, enriched with 2 proteins) and IL‐10 signaling pathway (from Reactome, enriched with 10 proteins). The IL‐10 anti‐inflammatory signaling pathway involving IL‐10 and HMOX1 was higher in HF, whereas the IL‐10 signaling pathway was lower in HF, led by 10 proteins (IL1RN, CCL22, CSF1, CCL20, IL10RB, IL12B, TIMP1, TNFRSF1B, ICAM1, and TNFRSF1A). This finding is surprising because IL‐10, IL10RB, ILRN, and CCL22 are generally considered anti‐inflammatory cytokines, while CSF1, CCL20, IL12B, ICAM1, and TNFRSF1A are proinflammatory cytokines or proteins involved in cytokine signaling. In IL‐10 pathways, IL‐10 binds to its receptor, initiating a cascade of events that ultimately leads to the suppression of inflammatory responses, primarily by inhibiting the production of proinflammatory cytokines and promoting immune tolerance, mostly via the Jak1/Tyk2 and STAT3 signaling pathway. A study by Voloudakis et al. reported a positive association of IL10RB with the COVID‐19 outcome severity; additionally, in vitro IL10RB overexpression is associated with increased viral load and activation of disease‐relevant molecular pathways (Voloudakis et al. 2022). These results suggest IL10RB may have a more complex role in immune regulation than previously understood. In fact, our finding underscores the potential significance of IL‐10 signaling pathway proteins in influencing the progression and severity of certain health conditions (Carlini et al. 2023) and in modulating the interplay between pro‐ and anti‐inflammatory proteins which may impact physical functions (Minuzzi et al. 2019).

Several lysophosphatidylcholines (LPCs) (16:0, 17:0, 18:0, 18:1 and 18:2) were significantly higher in HF compared to NF and LF (Figure 2C, right). Higher levels of LPCs, including 18:1 and 18:2, have been associated with higher mitochondrial oxidative capacity as well as better physical and cognitive function with aging (Tian et al. 2023). To test this association among participants, we created an “OXPHOS score” using 64 OXPHOS proteins from HF skeletal muscle data (Ubaida‐Mohien et al. 2022) (detailed in Methods) and analyzed its correlation with serum LPCs 18:0, 18:1, and 18:2. The correlation between serum LPCs 18:0, 18:1, and 18:2 and the skeletal muscle OXPHOS proteomic score showed a positive trend, but it was not statistically significant (Figure 2D, left). Intriguingly, the positive association between the skeletal muscle VO_2max_ and LPCs 18:0, 18:1, and 18:2 was more evident in HF and NF participants (*R* = 0.47, *p* = 0.003) (Figure 2D, right). LPCs are involved in the biosynthetic pathway of cardiolipin, a lipid unique to mitochondria and essential to the maintenance of the architecture of the cristae that in turn maintains the reciprocal spatial distribution of the respiratory complexes to ensure efficient transition of charged electrons (Drzazga, Sowinska, and Koziolkiewicz 2014; Semba et al. 2019a).

Interestingly, there was a corresponding higher level across LF to NF to HF in several phosphatidylcholines (PCs) (26/35) that may have contained fatty acids 18:0, 18:1, or 18:2, including 12/17 acyl PCs (PC aa) and 14/18 acyl alkyl PCs (PC ae) (Figure 2C, left). PCs are the major lipids distributed in the cell membrane and are enriched in lipid raft microdomains, which are the cholesterol‐rich regions of membranes where many cellular signaling proteins reside (Dean and Lodhi 2018). Accordingly, the majority of PCs were higher in HF compared to NF and LF, with the exception of long‐chain PC aa 38:4 and PC aa 40:4. The higher level of LPCs can also result from PCs via partial hydrolysis by the phospholipase (PLA2) activity. The LPC/PC ratio, reflective of PLA2 activity, was higher in LF (LF Beta = 4.5 and *p* = 0.017), compared to NF and HF, consistent with higher PLA2 activity (and consequently higher inflammation) in LF (Klavins et al. 2015; Sun et al. 2010). We observed higher glycine plasma levels in HF (HF beta = 0.467 and *p* = 0.035) compared to NF and LF (Figure 2E), which is consistent with its known anti‐inflammatory properties (Aguayo‐Ceron et al. 2023). Glycine upregulation is inversely related to systemic inflammation, and low plasma glycine levels have been associated with low‐grade inflammation and hepatic insulin resistance, obesity, and diabetes (Aguayo‐Ceron et al. 2023). Glycine can decrease proinflammatory cytokines and has been shown to modulate their production in innate cells (Aguayo‐Ceron et al. 2023) (Figure 2E). The kynurenine/tryptophan ratio (Kyn/Trp) was notably higher in LF compared to NF and HF (Figure 2F). The lower Kyn/Trp ratio in HF, despite the elevated Trp levels in HF, implies a shunted metabolism away from kynurenine production in HF. The Kyn/Trp ratio serves as a representative measure of systemic indoleamine 2,3‐dioxygenase‐1 (IDO1) activity (Moaddel et al. 2022; Westbrook et al. 2020), and heightened IDO1 activity is driven by cytokines such as IFN‐gamma, IFN‐alpha, and IFN‐beta (Wichers and Maes 2004), as well as by leptin (Figure S3) (Orlova, Shirshev, and Loginova 2019). Supporting this hypothesis, the Kyn/Trp ratio exhibited a significant and positive correlation with the “inflammatory score” (Figure 2A) (*R* = 0.5, *p* = 0.0015; Figure 2G).

Indole sulfate, which results from sulfonation in the liver, was lower in HF compared to NF and LF (HF beta = −0.125, *p* = 0.019), consistent with its proinflammatory role in macrophage activation (Nakano et al. 2019). Indole sulfate, a uremic toxin, and several other uremic toxins were lower in HF participants compared to NF and LF, including homocysteine and p‐cresol sulfate, suggesting decreased inflammation in HF compared to NF and LF (Figure 2H). In CKD patients, p‐cresol sulfate is associated with elevated levels of selected inflammatory mediators (Rossi et al. 2006). It is also noteworthy that uremic toxins have been shown to impair mitochondrial respiratory function by inhibiting matrix dehydrogenases in skeletal muscle in a mouse model of chronic kidney disease (Thome et al. 2019). Thus, the lower uremic toxin burden in serum of HF is consistent with the higher indices of mitochondrial function that we have reported previously in HF (Ubaida‐Mohien et al. 2022). The observed elevation in HCys levels among HF participants compared to NF and LF aligns with lower levels of cystathionine‐β‐synthase (CBS), which would result in lower levels of glutathione, a powerful antioxidant (Figure 2H).

### 2.3 Proteins and Metabolites Higher in Serum From LF Octogenarians Reflect Inflammation, Impaired NAD+ Metabolism, and Neurodegeneration

**FIGURE 3:**
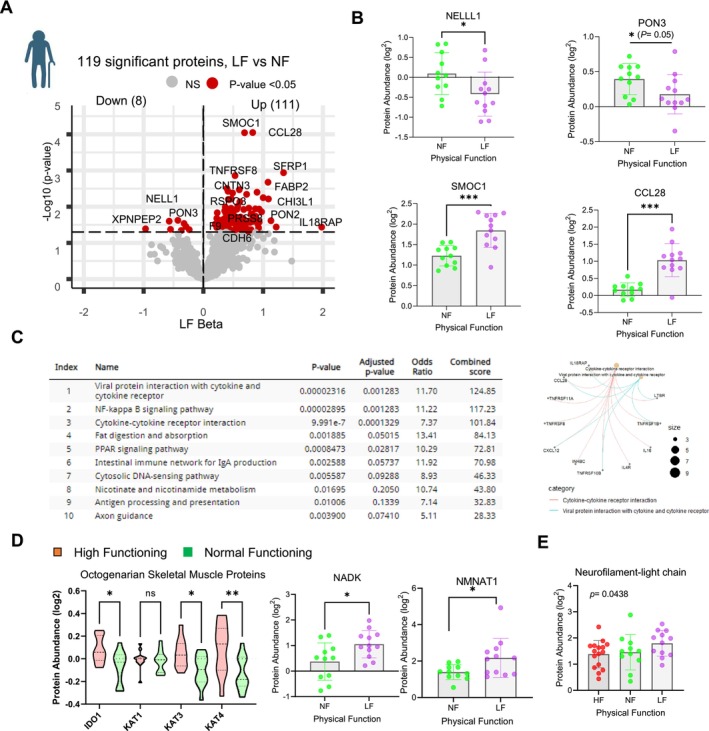
Proteins and metabolites higher in LF serum and associations reflect inflammation, impaired NAD+ metabolism, and neurodegeneration. (A) Number of significant proteins from LF serum proteome is shown, proteins higher in LF (right) and lower in LF (left) and effect size (*x*‐axis) are shown in the volcano plot. (B) Top significant LF proteins and log2 protein abundance are shown. (C) Top 10 significant pathways enriched in LF proteome from 119 LF proteins (*p* < 0.05) (left). Genes contribute to the top immune pathways, with shared and unique genes (right). (D) Log2 protein abundance of IDO, KAT1, KAT3, and KAT4 from HF and NF skeletal muscle participants. High NADK and NMNAT1 protein abundance shows a suggestive impaired NAD+ metabolism in LF. (E) NEFL abundance in LF participants. The *p*‐value significance of the metabolites measured from ANOVA.

When comparing LF to NF participants, there was an elevated level of proinflammatory proteins in the LF group. There were 119 dysregulated proteins in LF (Figure 3A, Table S2). SMOC1, the top protein that was higher in the LF group compared to NF, has an important role in intercellular matrix remodeling, but its function as a circulating protein is unknown (Montgomery et al. 2020). On the other hand, NELL‐1, the top protein with lower abundance in LF compared to NF (Figure 3B), is a potent growth factor that is highly specific to the osteochondral lineage (Zhang et al. 2010). Overall, enrichment analysis of the 119 differentially represented proteins in LF proteome reveals categories of cytokines and cytokine receptor proteins, inflammation‐related proteins, or DNA‐sensing proteins (DDX58). The most significantly enriched pathways are found in Figure 3C, along with the list of cytokine pathway genes (right). Some of the genes are shared among pathways, namely those related to cytokine receptor interaction and cytokine receptor pathways; however, each pathway was also driven by unique leading genes/proteins.

As mentioned above, our results highlight important differences in kynurenine metabolism according to the level of physical function. Kynurenine metabolism generates metabolites (Figure S4A) that are involved in the inflammatory/immune response (Miller 2013; Moaddel et al. 2022), several of which were measured in our study. Outside of the Kyn/Trp ratio which is representative of IDO activity, none of the kynurenine metabolites were significantly different. IDO1 is upregulated by proinflammatory cytokines including IL‐1β, IL‐6, IFN‐α, and TNF‐α (Heisler and O'Connor 2015; Wichers and Maes 2004). Leptin, which was also less abundant in HF serum (Figure S3), activates IDO1, which is the rate‐limiting enzyme for the conversion of Trp to Kyn, and likely contributes to the lower Kyn/Trp ratio in HF (Figure 3D). In addition, KAT3 and GOT2/KAT4, located in the mitochondria were also higher in the skeletal muscle proteome of HF (Figure 3D). These enzymes metabolize Kyn into kynurenic acid (KYNA) and help divert kynurenine metabolism away from the production of the cytotoxic quinolinic acid. This result suggests that HF may have better physical function in part because they better handle tryptophan metabolism or that higher physical function positively regulates kynurenine metabolism by promoting higher mitochondrial content (or both).

NAD+ is primarily synthesized in vivo through the salvage pathway, although *de novo* synthesis and the Preiss–Handler pathways also contribute (Demarest et al. 2019). The impact of the kynurenine metabolome on different PF groups suggests a potential influence on the *de novo* pathway, where dietary tryptophan is converted to NAD+. This is supported by the observed higher level of NADK (NAD+ Kinase) in LF serum compared to NF (Figure 3D). NADK is highly regulated by the redox state of the cell and regulates NADP+ synthesis in vivo. In the salvage pathway, NAMPT, a rate‐limiting enzyme, was not significantly different among different PF groups. NMNAT, another rate limiting enzyme in the salvage pathway that converts NMN to NAD+, was differentially abundant according to physical function groups. The mitochondrial NAD+ synthase (NMNAT3), which is generally highly expressed in skeletal muscle, was more abundant in the skeletal muscle of the HF group (Figure S4B), consistent with the reported increase in NAD+ levels in skeletal muscle in NMNAT3 Tg mice (Gulshan et al. 2018). Surprisingly, in serum, the nuclear isoform, NMNAT1, which plays a role in nuclear NAD+ homeostasis, was more abundant in LF (Figure 3D). The higher circulating levels of NMNAT1 may represent a compensatory response aimed at increasing NAD+ levels and would be consistent with the concomitant higher level of NADK observed in the LF group.

The results suggest that poor physical function is associated with the impairment of the pathways that convert dietary tryptophan to NAD+ and other metabolites, possibly due to the proinflammatory state. In particular, serum NAD+ kinase (NADK), an enzyme that phosphorylates NAD+ producing nicotinamide adenine dinucleotide phosphate (NADP), was significantly lower in LF compared to HF. There is strong evidence that NADK is an essential modulator of cellular redox homeostasis and metabolism in multicellular organisms, and a lower level may be involved in neurodegeneration (Oka et al. 2023). Since experiments in the model organisms of NMNAT overexpression show a neuroprotective function of NMNAT, the higher serum levels of NMNAT1 in LF may represent a compensatory response to neurodegeneration in this group (Zhai et al. 2008). Consistent with this idea, the LF group had a higher level of the neurodegeneration biomarker NEFL (Brousse et al. 2023; Chatterjee et al. 2019; Khalil et al. 2020) compared to the other groups (Figure 3E).

### 2.4 High Physical Function Is Associated With Serum Protein Profile Indicating Blunted Inflammation

**FIGURE 4:**
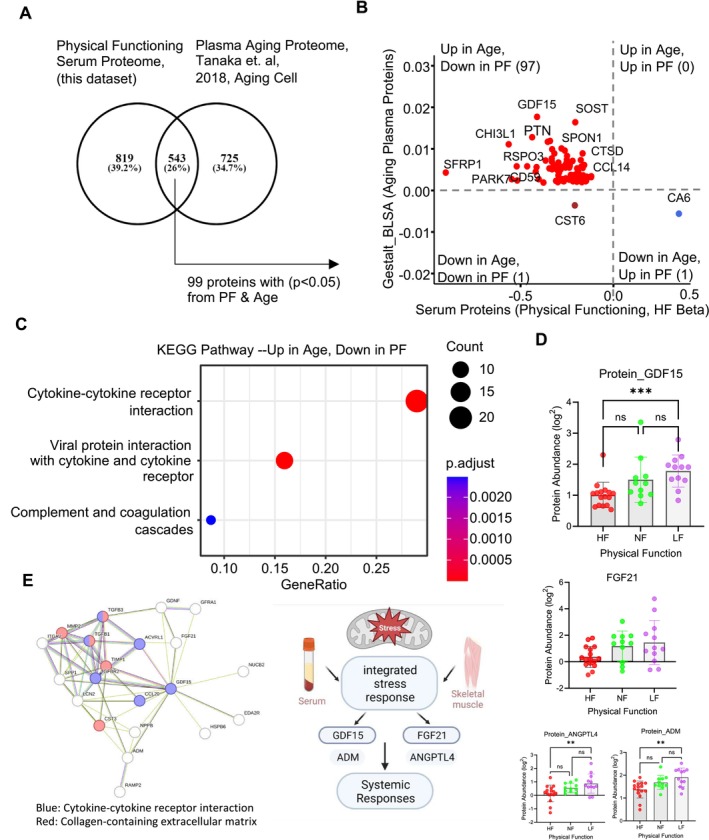
High physical function is associated with serum protein profile indicating blunted inflammation. (A) Comparison between serum proteome (this dataset) and plasma aging proteome (Tanaka et al. 2018). 543 common proteins between datasets and 99 significant proteins (*p* < 0.05) changed with PF and age. (B) Representation of 99 PF and age proteome, red circles are proteins up in plasma aging study and down in PF octogenarians. (C) Proteins discordant between PF and age were analyzed for pathway enrichment showed immune and inflammatory pathways suggesting a blunted inflammation in PF compared to aging proteome. (D) Most significant gene from this enrichment analysis is shown (GDF15). (E) Network of proteins interacting with GDF15 showed FGF21 and other proteins for mitokine homeostasis and ISR. Protein abundance of GDF15‐related proteins.

Given that declining physical function is a hallmark of normal aging (Alcazar et al. 2023), we examined how serum biomarkers associated with physical function related to circulating biomarkers of aging. The serum PF proteome was compared with the aging plasma proteome published previously by Tanaka et al. (2018), a study which involved 240 healthy participants aged 22–93 years (Figure 4A). We analyzed significant proteins (99 proteins, *p* < 0.05) common between the PF proteome and the aging plasma proteome. Interestingly, 97 proteins whose levels increase with age (aging plasma proteome) were lower in the PF proteome, suggesting that high physical functioning and aging processes act in opposite directions (Figure 4B). These discordant proteins were lower in HF individuals and increase with the aging process. An enrichment analysis of these 97 proteins that increase with aging revealed an over‐representation of inflammatory protein pathways. This implies a blunted inflammation in HF, compared to the overall plasma aging proteome (Figure 4C). Due to the cross‐sectional nature of our study, it is not possible to disentangle the positive impact of higher physical activity from a beneficial gene–environment predisposition on the inflammatory state in HF, but both factors are likely influential here.

Interestingly, enrichment analysis highlights GDF15 (HF Beta = −0.41, *p* = 0.0002) (Figure 4D), and its interaction with other mitokines, including FGF21, which was less abundant in HF compared to NF and LF (HF Beta = −0.534, *p* = 0.025) (Figure 4E). Mitokines are mediators of some metabolic and mitochondrial stress responses (Figure 4E). GDF15 and FGF21 have been described as mediators in the resilience arm of the integrated stress response (ISR), a stress response mechanism that can be activated by multiple stressors, including mitochondrial dysfunction. While they reduce protein translation, they also enhance a wide range of defensive mechanisms, including proteostasis (Pakos‐Zebrucka et al. 2016). For example, high production of FGF21, a cytokine with both paracrine and endocrine functions, has been shown to be induced by respiratory chain dysfunction (Croon et al. 2022). Moreover, GDF15 is a powerful risk factor for cardiovascular disease and other adverse health outcomes such as disability and mortality (Daniels et al. 2011; Wollert, Kempf, and Wallentin 2017) and is also known to be elevated in children with primary mitochondrial disease (Montero et al. 2016). However, a recent analysis in the UK biobank that contrasted circulating proteins associated with mortality estimated from Mendelian randomization found that GDF15 is both a biomarker and a causal effector in chronic disease‐related mortality and that GDF15 may exhibit differing and even opposing functions depending upon the metabolic, developmental, and tissue‐specific circumstances (Sethi et al. 2024). Acknowledging this complexity in the interpretation of differences in GDF15 levels, the higher GDF15 in LF could reflect a higher degree of mitochondrial stress and a biological compensation to try to prevent it from spiraling out of control.

### 2.5 High Physical Function Is Associated With Lower Evidence of Inflammatory Factors Produced by Senescent Cells

**FIGURE 5:**
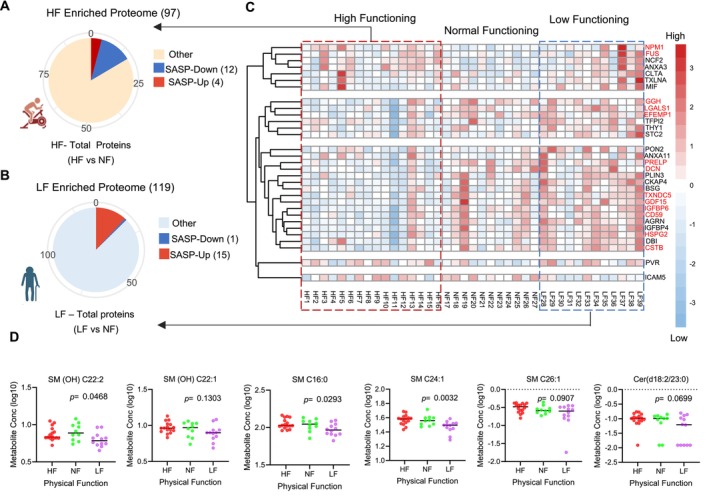
High physical function is associated with lower evidence of inflammatory factors produced by senescent cells. (A, B) Senescence‐associated secretory proteins (SASP) from HF and LF using SASPAtlas (Basisty et al. 2020). 16% and 14% of the significant HF and LF proteins were associated with SASP, respectively. 12/16 SASP protein abundance was lower in HF (higher levels for NCF2, ANXA3, NPM1, and FUS) while ICAM5 was lower in LF. (C) All significant SASP proteins from HF and LF (top right) were shown in the heatmap plot. HF significant SASP proteins are in red font. Abundance of the SASP protein is color‐coded, showing that LF has higher SASP protein levels compared to NF. Lower levels of proteins is coded as blue and higher levels as red. (D) Abundance of sphingomyelins and ceramides in HF, NF, and LF participants. The *p*‐value significance of the metabolites obtained from ANOVA.

Cellular senescence stands as one of the fundamental cellular mechanisms at the interface between the aging process and the development of tumors. There is some evidence that senescent cells accumulate with aging and produce a prolonged low‐level inflammation (Ovadya et al. 2018), abnormal tissue growth, and intercellular matrix changes through the secretion of senescence‐associated secretory phenotype (SASP) factors (Li et al. 2023). It is noteworthy that 16% of the differentially represented HF serum proteins and 14% of the LF serum proteins have previously been identified as SASP proteins (Basisty et al. 2020). The abundance of SASP proteins from HF, NF, and LF is illustrated in Figure 5C. Interestingly, 75% of these proteins (12 in total) were lower in HF (Figure 5A, left), while conversely, 94% of SASP proteins were higher in LF (Figure 5B, left). To note, intercellular adhesion molecule 5 (ICAM5), a molecule reported to have a role in regulating gene expression and histone modification process (Crossland et al. 2017), was lower in LF (Figure 5C). Moreover, Tian et al. (2008), reported that neuronal ICAM5 is involved in immune privilege of the brain and acts as an anti‐inflammatory agent. Comparison between LF and NF groups showed a negative association of IL6 (LF Beta = 0.820, *p* = 0.026) in the LF group, while ICAM1 (HF Beta = −0.131, *p* = 0.039) and VCAM1 (HF beta = −0.176, *p* = 0.006) were lower in HF compared to NF and LF. Major cytokines and growth factors including IL‐6, ICAM, VCAM, etc. were reported as SASP by in vivo and in vitro analyses by radiation‐induced senescence from rat glomerular endothelial cells (Aratani et al. 2018). The secreted IL‐6, IL‐1, IL‐8, and VEGF were reported to further amplify cellular senescence in neighboring cells through a paracrine effect (Shakeri et al. 2018). These results might suggest that either the physical activity regimen of octogenarian HF master athletes may prevent the accumulation of cellular senescence in tissue or rather indicate a lower predisposition to the accumulation of cellular senescence that is permissive to the conservation of a high level of physical function (or both). There is some evidence that exercise promotes a healthier cellular milieu that counteracts the senescence program and facilitates immune cell–mediated senescent cell clearance (Zhang et al. 2022). The metabolomic data also support higher senescence in LF, as observed by the perturbed sphingomyelin/ceramide pathways (Moaddel et al. 2024; Trayssac, Hannun, and Obeid 2018). There was a higher abundance of sphingomyelins and ceramides in HF participants compared to NF and LF (Figure 5D). The low abundance of these metabolites in LF may suggest lower levels in sphingosine and sphingosine‐1 phosphate (S1P) in LF participants, resulting in lower S1P levels promoting cell survival and proliferation of these metabolites (Moaddel et al. 2022).

### 2.6 Octogenarians With High Physical Function Have Serum Protein Profile Consistent With Less Denervation

**FIGURE 6:**
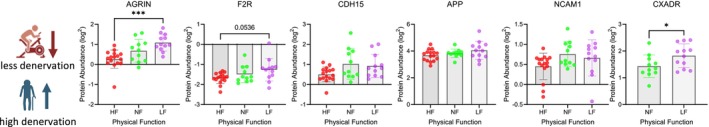
Octogenarians with high physical function have serum protein profile consistent with less denervation. Denervation‐related protein abundance in HF suggesgted less denervation in HF, but more denervation in LF. CXADR, key regulator of adhesion and inflammation, was more abundant in LF compared to NF. The *p*‐value significance of the proteins is shown as (*p < 0.05), (***p < 0.001) measured from ANOVA and Kruskal–Wallis test.

Aging is associated with progressive denervation of muscle fibers, which is not fully compensated by reinnervation and leads to progressive fiber atrophy that culminates in a loss of muscle fibers (Hepple and Rice 2016). There is evidence that the level of persistent denervation is substantially lower in older persons who maintain high physical function (Sonjak et al. 2019). Older persons who are highly physically active also show greater fiber‐type grouping and greater muscle mass (Mosole et al. 2014). Agrin (AGRN) is produced by motor neurons and when bound by Lrp4 results in the activation of MuSK to induce the aggregation of nicotinic acetylcholine receptors at the endplate and contributes essentially to the reformation of the neuromuscular junction following denervation (Tintignac, Brenner, and Ruegg 2015). Serum levels of AGRN, particularly the cleaved agrin fragment (CAF), has been shown to be higher in elderly with low muscle mass (Pratt et al. 2024) and is thought to reflect the burden of denervation in skeletal muscle (Hettwer et al. 2012). Serum AGRN was significantly lower in HF compared to NF and LF, suggesting that HF is characterized by a lower level of denervation (Figure 6). A high level of AGRN in LF suggests a high level of denervation and incomplete reinnervation, as can occur in sedentary and frail older persons (Sonjak et al. 2019). Furthermore, CXADR, a protein which is enriched at the neuromuscular junction (Shaw et al. 2004), was higher in the serum of LF versus NF, suggesting it may also reflect a higher burden of denervation in LF. These differences among HF and NF, and LF and NF, are generally consistent with previous studies suggesting that muscle mass and function with aging are related to the capacity for reinnervation (Piasecki et al. 2018).

## 3 Discussion

Aging is associated with a greater risk for chronic conditions, as well as physical and cognitive functional decline (Walker et al. 2022). However, there is a high degree of heterogeneity in health and function with chronological age. Thus, while a large proportion of people in their 8th and 9th decades of life are affected by chronic diseases and impairment, with some being severely frail, a small fraction, such as master athletes, have lower chronic disease burden and maintain exceptional physical and cognitive capacity. It is accepted that genetic, environmental, and behavioral factors all contribute to the chance of living a long, healthy, and active life. For example, an active lifestyle or regular exercise regimen is associated with an attenuated decline of physical function (Skoglund et al. 2023; Soendenbroe et al. 2020; Ubaida‐Mohien et al. 2019; Voisin et al. 2023) and also benefits cognitive function (Kim, Kwon, et al. 2020; Kim, Han, et al. 2020; Zhao et al. 2016) with aging, but the underlying mechanisms remain under investigation.

**FIGURE 7:**
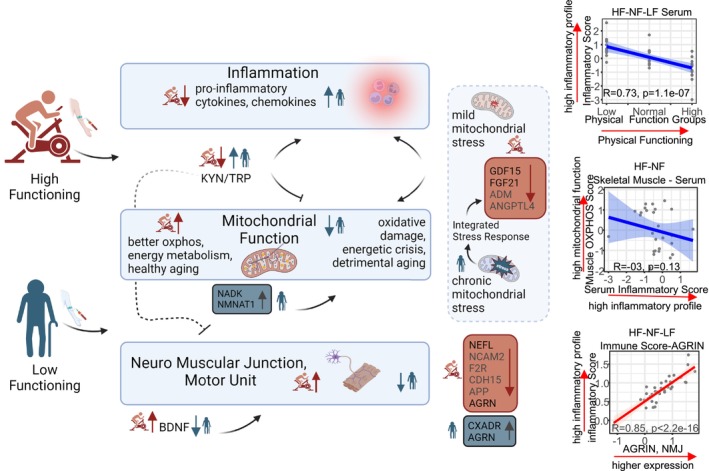
Mechanistic model of inflammation, mitochondrial dysfunction, and neuromuscular junction characteristics of HF and LF octogenarians. (Right). Each participant has an inflammatory score assigned from 114 immune pathway proteins correlated with the physical functioning group, which showed a high pro‐inflammatory profile in LF. Skeletal muscle OXPHOS score correlation with serum inflammatory score is shown. HF octogenarians have low inflammatory profile with better OXPHOS score while NF has low OXPHOS function and high inflammatory profile. Higher abundance of Agrin associated with higher inflammatory profile. Each participant is represented as a gray circle and a blue/red regression line with confidence interval. Spearman's rank correlation *p*‐value is shown at the bottom of the graph.

To explore potential physiological mechanisms underlying different health and functional status in old age, we compared serum proteins and metabolites among a small cohort of high‐functioning octogenarian master athletes (HF), healthy normal‐functioning octogenarian non‐athletes (NF), and low‐functioning octogenarian non‐athletes (LF). We found that a higher performance status was associated with: (1) lower levels of circulating proinflammatory markers, as well as a corresponding lower level in IDO activity associated with the metabolic evidence of normal tryptophan metabolism, with the normal function of the kynurenic pathway; (2) higher circulating levels of LPCs that have been associated with better mitochondrial oxidative capacity; (3) a lower degree ISR activation; (4) lower levels of circulating proteins that have been identified as SASP members; and (5) lower level of proteins that reflect neurodegeneration/denervation (Figures 6 and 7).

The notion that healthy aging is associated with a lower level of a proinflammatory state is well established in the literature (Picca et al. 2023) and is supported in this study. We found that not only are proinflammatory markers significantly higher in older persons with lower physical function but also that there is a continuum of increasing inflammation severity from HF to NF to LF (Figure 7B), although the proinflammatory markers implicated appear to be slightly different within the different function groups. For example, IL6 was significantly higher in the LF group compared to NF, while ICAM1 and VCAM1 were lower in HF, compared to NF. These findings suggest that different aspects of inflammation should be considered across the spectrum of physical function, and understanding such differences may reveal important nuances in the relationship between inflammation and physical function.

We have previously shown that the vastus lateralis muscle proteomics of HF octogenarians are enriched with mitochondrial structure and function proteins, particularly those pertaining to electron transport capacity (ETC), cristae formation, mitochondrial biogenesis, and mtDNA‐encoded proteins (Ubaida‐Mohien et al. 2022). Recent literature points to mitochondria as the central hub of chronic inflammation (Marchi et al. 2023). Dysfunctional mitochondria can release oxidized mitochondrial DNA and oxidized cardiolipin that trigger the three major pathways to inflammation, namely, the NF‐kB, the NLRP3 inflammasome, and the cGAS‐STING pathways (Singh et al. 2023; Walker et al. 2022). Therefore, a plausible hypothesis is that a high level of physical function, by promoting mitochondrial health, may downregulate inflammation through multiple pathways (Figure 7). Interestingly, a recent study suggests that in senescent cells, dysfunctional mitochondria release mtDNA into the cytosol and through activation of the cGAS–STING pathway enhance the production of SASP (Victorelli et al. 2023). This notion is consistent with the findings of higher levels of SASP proteins in the LF individuals compared to the HF and NF.

This analysis also supports a direct connection between inflammation and metabolism. The significant differences in cystine, Kyn/Trp ratio (IDO activity), and homocysteine levels across functional levels are remarkable. In addition to the lower Kyn/Trp ratio in HF (Figure 2F), a significant correlation between Kyn/Trp and inflammatory score (Figure 2G) was also observed. This is consistent with the notion that immunosenescence is associated with a proinflammatory state but reduced immunocompetence (Singh et al. 2023). The results also provide additional evidence for a connection between inflammation and tryptophan metabolism. We found that high functioning was associated with higher KAT3 and GOT2/KAT4, enzymes that metabolize Kyn into kynurenic acid (KYNA) and help divert Kyn metabolism away from the production of the cytotoxic quinolinic acid. The findings of this study are also consistent with the data in the literature showing that higher levels of long‐chain LPCs are associated with a higher mitochondrial function assessed by ^31^P‐spectroscopy and less decline of mobility over time (Gonzalez‐Freire et al. 2019; Semba et al. 2019b). The role of LPCs on cardiolipin synthesis, mitochondria‐specific lipid composition, and the maintenance of the mitochondrial architecture and function and may be, at least in part, the explanation for our findings.

In this study, we found in LF compared to NF participants higher levels of serum proteins that have been described as members of the SASP. Many of these proteins appear to be increasing with aging in the general population (Schafer et al. 2020; Tanaka et al. 2018), suggesting that a high level of physical function is associated with the lower accumulation of senescent cells. Remarkably, differences were particularly evident in the comparison between LF and NF, and substantially less in the comparison between HF and NF. This finding suggests that the accumulation of senescent cells mostly occurs in people who are frail and/or have a low level of physical function. Accumulation of senescent cells increases SASP release, including cytokines, metalloproteinases, and DAMPs into circulation, that may aggravate the inflammatory status (Walker et al. 2022).

The lower level of mitokines, such as FGF21 and GDF15, in HF participants, combined with the evidence for better mitochondrial function we reported previously in this HF group (Ubaida‐Mohien et al. 2022) is consistent with the evidence that FGF21 and GDF15 reflect mitochondrial stress (Ito et al. 2018; Lehtonen et al. 2016). Stress response proteins increase during the conditions of energetic challenge, although biologically, they have been shown to have potential anti‐aging effects by promoting cellular health and metabolic homeostasis. For example, GDF15 and FGF21 levels increase dramatically in response to strenuous exercise (Campderros et al. 2020). Thus, the data showing lower levels of mitokines in HF individuals and higher levels in LF could suggest a lower level of mitochondrial stress in HF than LF (Figure 7). Our study is consistent with the evidence that increased expression of denervation‐responsive genes was negatively associated with physical function in elderly men and women (Lukasiewicz et al. 2023). The higher abundance of NEFL in the LF group may reflect an increased burden of denervation, consistent with previous studies finding that NEFL levels are elevated in populations with neuromuscular disease (Brousse et al. 2023; Sandelius et al. 2018) and in aged individuals with more severe muscle loss (Pratt et al. 2022). Further to this point, the lower levels of AGRN in the HF group suggest a potential mitigation of muscle denervation, consistent with neuromuscular junction integrity playing an important role in physical function with aging (Figure 7). However, this hypothesis should be further tested in longitudinal studies in the future. In summary, this study underscores the multiple pathways that distinguish higher versus lower physical function in older individuals. The extent to which these circulating factors can be modulated by regular physical activity should be examined in longitudinal studies and ultimately tested in randomized clinical trials.

### 3.1 Limitations

While this study provides valuable insights into differences in serum proteomics among participants with contrasting physical function, confirmation in a larger cohort will be important in future studies. It is also important to acknowledge the different geographical residences of the participants, where the HF group were from Canada, the US, and the UK; the LF group were from Montreal Canada, Gainesville FL, USA, Pittsburgh PA, USA, and Winston‐Salem NC, USA; and the NF participants were from Montreal Canada. Thus, there could be dietary, lifestyle, or other environmental influences, particularly for the NF group who were entirely from the Montreal area, that affected the serum constituents. Furthermore, longitudinal studies are warranted to elucidate the dynamic relationships between physical activity, molecular pathways, and aging. In this respect, it is also important to acknowledge that the serum measures represent a single measurement in time and thus cannot provide insights into dynamic changes related to flux of specific pathways that may be more directly pertinent to function. It is also clear that differences in the amount and quality of physical activity among groups will contribute to the proteomic and metabolic differences observed, although it is important to stress that the nature of the blood collection (at least 48 h since last bout of physical activity) would rule out an acute effect of exercise. Further to this point, 15 of the HF participants were ranked in the top 3 worldwide athletic events for their age group and discipline, meaning that their exercise habits alone are unlikely to explain their high function. Diet is another factor that was not controlled for in this analysis and could contribute to differences among groups. Disentangling the impact of physical activity habits from genetic and environment differences among groups is simply not possible with this study design. Finally, the limited number of proteins and targeted metabolites quantified in this study may not capture the full spectrum of the molecular landscape associated with physical function in octogenarians.

## 4 Methods

### 4.1 Study Participants and Serum Sampling

Study participants were recruited as part of a study of octogenarian competitive athletes as a model for healthy aging at McGill University (all high‐functioning and normal‐functioning participants and three low‐functioning participants), a Pepper Pilot Study at the University of Florida (one low‐functioning participant), and The Study of Muscle, Mobility, and Aging (SOMMA) (eight low‐functioning participants). The high‐functioning participants were recruited from international track and field and cycling masters athlete competitions and represented athletes from Canada, the US, and the UK. Approval by the relevant institutional review boards was obtained at each site (McGill Faculty of Medicine IRB: A08‐M66‐12B; University of Florida: IRB201801384; SOMMA: WCG IRB 20180764). The normal‐functioning participants were recruited from the Montreal (Canada) area and were non‐athletes, approximately half of whom engaged in some regular physical activity such as golf, walking, or “heavy gardening”. All normal‐functioning participants were independent‐living with an SPPB score > 8. SOMMA is a prospective cohort study of mobility in community‐dwelling older adults, where participants for the current study were from the baseline cohort, enrolled between April 2019 and December 2021 and represented individuals recruited from Pittsburgh PA and Winston‐Salem NC. Additional details of the SOMMA cohort have been published previously (Cummings et al. 2023). All participants provided written informed consent. Additional details of the participants by group are provided in Table 1, noting that inclusion in the low‐functioning group was based upon SPPB < 8. The 4 m walk test used to establish gait speed for all participants at all sites was performed according to the protocol by Vasunilashorn et al. (2009). Blood samples for all participants were obtained from an antecubital vein after an overnight fast of at least 8 h and at least 48 h following the last bout of physical activity. There was no difference in the age or sex distribution among groups. Serum blood samples from 16 HF (mean age 80.2, 50% male, 8 of whom were world record holders in their discipline at the time of testing), 11 NF (mean age 80.5, 63% male), and 12 LF (mean age 78.5, 42% male) were analyzed for metabolites and proteins as detailed below.

### 4.2 Proximity Extension Assay Proteomic Analysis

Serum proteins were measured using Olink Explore 1536 (Olink Proteomics AB, Uppsala, Sweden) according to the manufacturer's instructions (www.olink.com) and as previously described (Moaddel et al. 2023). The technology behind the Olink protocol is based on Proximity Extension Assay (PEA) (Assarsson et al. 2014), coupled with readout via next‐generation sequencing (NGS). The raw output data are converted into Normalized Protein eXpression (NPX). Data normalization was performed using an internal extension control and an external plate control to adjust for intra‐ and inter‐run variation as previously described (Moaddel et al. 2023). All assay validation data (detection limits, intra‐ and inter‐assay precision data, predefined values, etc.) are available on the manufacturer's website.

### 4.3 Serum Metabolomic Assay

A targeted metabolomic assessment was carried out following the manufacturer's protocol using Biocrates' MxP Quant 500 kit (Biocrates, Innsbruck, Austria), which quantifies 630 distinct metabolites in human plasma (10 μL) (Moaddel et al. 2022). Briefly, metabolites were measured using a Nexera HPLC system (Shimadzu) coupled to a 6500+ QTRAP mass spectrometer (AB Sciex) with an electrospray ionization source, as previously described (Moaddel et al. 2022). Data were quantified using appropriate mass spectrometry software (Sciex Analyst) and imported into Biocrates MetIDQ software for further analysis. The data were normalized to internal quality controls.

### 4.4 Kynurenine Metabolome

Separation of the kynurenines (Kyn) was accomplished as previously described (Westbrook et al. 2020). Briefly, to 40 μL plasma, 10 μL internal standard and 10 μL 0.1% formic acid in water were added and loaded on solid‐phase extraction cartridges (Oasis HLB, Waters Corp.). The metabolites were eluted with 1 mL of 0.1% formic acid in 95:5 methanol/water and stream‐dried under nitrogen. The samples were reconstituted in 100 μL of 0.1% formic acid in 10:90 methanol/water and transferred to autosampler vials for analysis. Separation of the KYNs was accomplished using a linear gradient (0–1 min 5% B, 3 min 23% B, 3.1–5 min 70% B, 5.5–20 min 90% B, 20.1 min 10% B, and 21 min 5% B) at 0.3 mL/min for 30 min at 40°C, on an X‐Select HSS C18 column (2.1 × 150 mm, 2.5 μm, Waters), with mobile phase A consisting of 0.2% aqueous formic acid and mobile phase B consisting of 0.2% formic acid in methanol. Data were acquired using a Nexera XR HPLC system (Shimadzu) coupled with a QTRAP 6500+ (SCIEX) instrument and were analyzed with Analyst 1.6 (SCIEX). Relative concentrations of the metabolites were determined in standard solution using area ratios calculated using a deuterated standard; because matrix effects were not considered, these only provide a measure of relative abundance and not absolute quantification. The data were normalized to a pooled sample of study participants.

### 4.5 Data Processing, Bioinformatics, and Statistics

Normalized serum proteomics and metabolomics data were filtered, log2‐transformed for further analysis. Proteins or metabolites were excluded from analysis if they were not present in ≥ 30% of the subjects (LOD). Data missing in < 30% of the subjects were imputed with 1/5 of the minimum positive value of each variable. A multivariable linear model was used to evaluate the linear change among the physical functioning groups (HF, NF, and LF), and also two other linear models were constructed to assess differences between HF and NF, and between LF and NF. Linear models were adjusted for age and sex. R build‐in lm() was used for the model design, *p*‐value derived from the model was considered significant (*p* < 0.05), and the significant proteins and metabolites were used for further downstream analysis. The results from these linear models were termed as PF proteome (HF, NF, and LF), HF proteome (HF vs. NF), and LF proteome (LF vs. NF). The same analysis strategy was implemented for metabolites as well. Linear model metabolite results were termed as PF metabolome (HF, NF, and LF), HF metabolome (HF vs. NF), and LF metabolome (LF vs. NF). To account for multiple comparisons, *p*‐values were corrected by the Benjamini–Hochberg procedure to control the false discovery rate and reported in the Supporting Information. Calculated *p*‐values (*p*) for physical function comparisons and effect size (β physical function (HF or LF) similar to log2 fold‐change) are indicated in the figure panels or legends. All statistical analyses were conducted using R (4.2.3) with built‐in libraries and functions.

Two score methods were implemented: Inflammatory score and OXPHOS score. For immune pathway inflammatory score, log_2_ abundance of all immune proteins from the serum HF pathway proteomic profile (Figure 2A proteins) was used (average of immune proteins divided by the total number of immune proteins) for each participant. The score was further used to correlate with the physical functioning groups. HF‐NF skeletal muscle data were used to calculate the OXPHOS score; 64 OXPHOS proteins reported by Ubaida‐Mohien et al. 2022 were used to measure the OXPHOS score for each participant (the average abundance of OXPHOS proteins divided by the total number of OXPHOS proteins). The list of proteins used for the score is reported in Table S3. Volcano plots for proteins/metabolites and heatmap to visualize protein/metabolite abundance results were generated using the base R plot function and libraries such as ggplot2 (3.5.0), enhanced volcano plot (1.16.0), complex heatmap (2.14.0), clusterProfiler (4.6.2), and GraphPad Prism (9.3.0). Robust correlations (*pbcor*) and Spearman's correlation were used to assess the relationship between the physical function and protein or metabolite. Protein and metabolite functional information were determined with the literature research for clinical and functional analyses. Gene ontology enrichment analysis was performed by ClusterGO and Enrichr. To identify biological functions and pathways that were enriched in the group analysis, an Over‐representation Analysis (ORA) was performed in Enrichr tool. Significant pathways or functional categories from the pathway enrichment analysis results of KEGG, Reactome, and Wiki database (2023) were selected. Proteins or a set of proteins were significantly enriched/depleted within a particular biological pathway or functional category (than would be expected by chance), indicating “over‐representation” or “under‐representation” of that pathway within the selected data subset. Interaction network of protein aalysis was performed by STRING functional protein association networks and Reactome database (2023). Network visualizations were performed in Cytoscape (3.8.0).

Considering the significant impact of physical function on the aging process, we aimed to examine the broader systemic effects of physical function in relation to aging. To achieve this, we utilized two additional datasets alongside the serum analysis: A skeletal muscle dataset obtained from the same HF and NF subjects (Ubaida‐Mohien et al. 2022) and an aging plasma dataset previously published by Tanaka et al. (2018). Total proteins and significant proteins (*p* < 0.05) reported in the respective manuscripts have been used in the analysis.

## Author Contributions

L.F. and R.T.H. conceived and designed the study. T.T., P.M.C., and J.A.M. collected the muscle biopsies, and S.S., N.J.M., L.F.F., and M.‐E.F. performed phenotypical analysis of the data. C.U.‐M. and R.M. designed the Olink proteomics and metabolomic assays; R.M. generated and preprocessed the data. J.C. supported the statistical analysis. C.U.‐M., R.M., R.T.H., and L.F. analyzed the data and prepared the figures. C.U.‐M., R.M., R.T.H., and L.F. wrote the manuscript, and all authors discussed the results and reviewed the final manuscript.

## Ethics Statement

Study participants were recruited as part of a study of octogenarian competitive athletes as a model for healthy aging at McGill University (all high‐functioning and normal‐functioning participants, three low‐functioning participants), a Pepper Pilot Study at the University of Florida (one low functioning participant), and The Study of Muscle, Mobility, and Aging (SOMMA) (eight low‐functioning participants). The high‐functioning participants were recruited from international track and field and cycling masters athlete competitions. Approval by the relevant institutional review boards was obtained at each site (McGill Faculty of Medicine IRB: A08‐M66‐12B; University of Florida: IRB201801384; SOMMA: WCG IRB 20180764). All human subjects research was done according to the Declaration of Helsinki.

## Consent

All subjects provided written informed consent.

## Conflicts of Interest

The authors declare no conflicts of interest.

## Code Availability

R codes and standard statistical analysis were carried out using R Studio (2023.06.0 Build 421). Data have been analyzed using existing standard packages and scripts described in Methods. No new code has been developed. Implementation of standard R libraries and functions can be found elsewhere at https://www.rstudio.com/products/rpackages/.

## Supporting information




**Figure S1.** Participant characteristics (walking speed, grip strength, and grip strength by sex and VO_2peak_) representation of HF, NF, and LF groups.
**Figure S2.** Comparison of PF, HF, and LF proteomes.
**Figure S3.** Protein abundance of leptin across PF, HF, and LF proteomes.
**Figure S4.** Simplified illustration of the kynurenine pathway (KP) and the metabolites quantified in the pathway from all samples (*n* = 38). Yellow borders are metabolites below the LOD.
**Figure S5.** OXPHOS score of the NF and HF skeletal muscle groups.

**Table S1.** Significant proteins quantified for PF, HF, and LF proteomes.

**Table S2.** Significant metabolites quantified for PF, HF, and LF metabolomes.

**Table S3.** Pathway and immune score proteins from serum and skeletal muscles.

## Data Availability

Skeletal muscle data used for this project are available in MassIVE with the dataset identifier MSV000086195 (https://massive.ucsd.edu/ProteoSAFe/dataset.jsp?accession=MSV000086195). All proteomic and metabolomic data supporting the findings are included in the manuscript as supplementary files. All raw data associated with this study and participant phenotypic information on the data can be obtained upon request.
