# Supplementary material for: Serum Proteomic and Metabolomic Signatures of High Versus Low Physical Function in Octogenarians
Source: Aging Cell. 2025 Mar 10;24(5):e70002. doi: 10.1111/acel.70002 (PMC12073904; doi:10.1111/acel.70002)

Supplementary Figures

Serum Proteomic and Metabolomic Signatures of High versus Low Physical Function in Octogenarians

Supplementary Figure 1

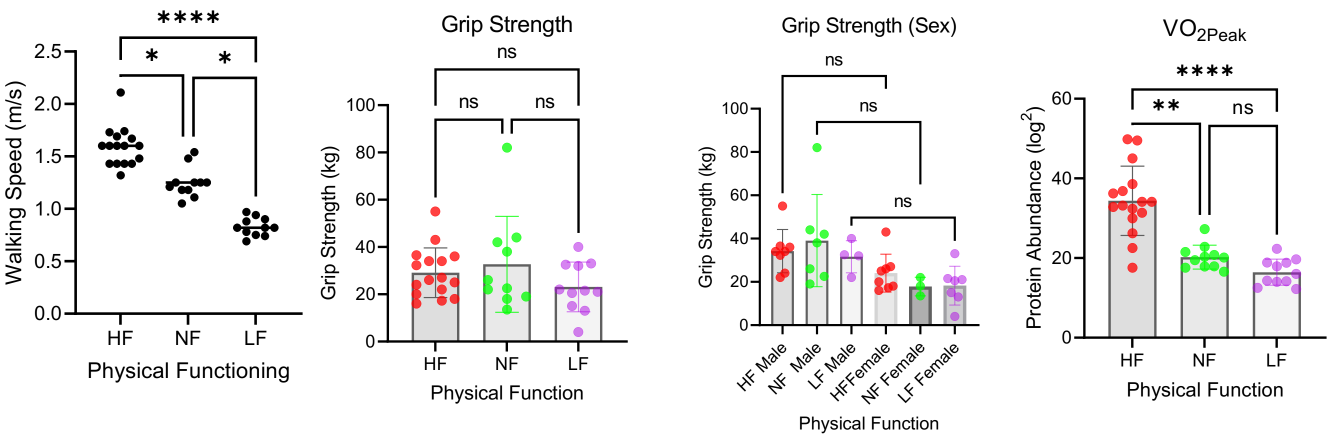

Supplementary Figure 2

A

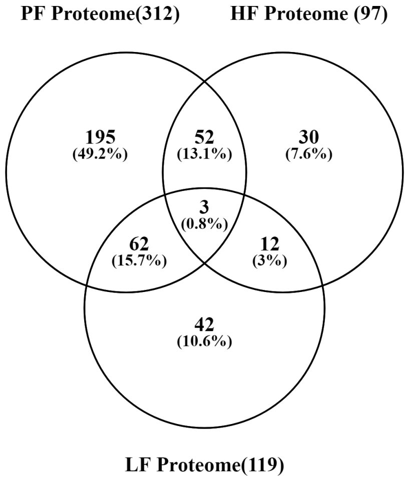

B

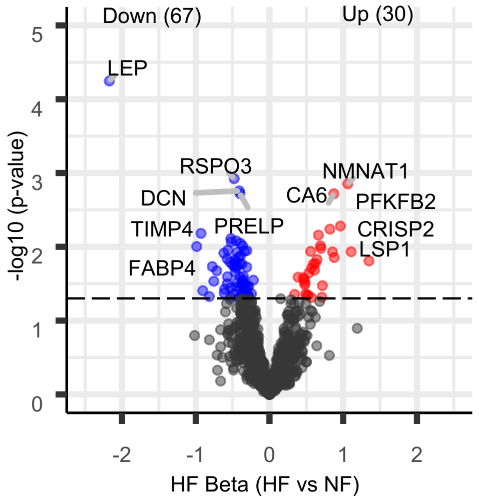

Supplementary Figure 3

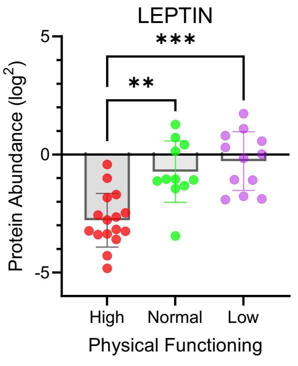

Supplementary Figure 4

A)

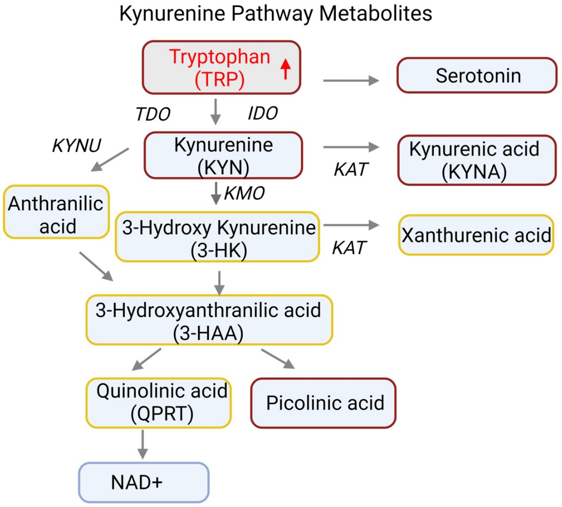

Kynurenine Pathway Metabolites Quantified

Metabolites (HMDB)	Effect Size Low Functioning	p-value
Kynurenine (HMDB0000684)	0.044	0.071
Kynurenic Acid (HMDB0000715)	0.002	0.067
Picolinic Acid (HMDB0002243)	-0.004	0.747
Tryptophan (HMDB0030396)	-2.172	0.001
Serotonin (HMDB0000259)	0.007	0.549

B)

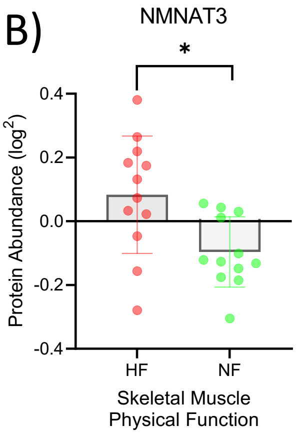

Supplementary Figure 5

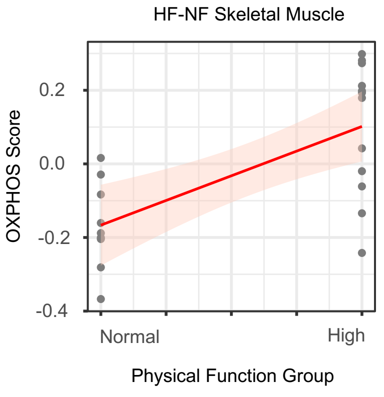

Supplement: Supplementary file 1 — Figure S1. Participant characteristics (walking speed, grip strength, and grip strength by sex and VO2peak) representation of HF, NF, and LF groups. Figure S2. Comparison of PF, HF, and LF proteomes. Figure S3. Protein abundance of leptin across PF, HF, and LF proteomes. Figure S4. Simplified illustration of the kynurenine pathway (KP) and the metabolites quantified in the pathway from all samples (n = 38). Yellow borders are metabolites below the LOD. Figure S5. OXPHOS score of the NF and HF skeletal muscle groups. [file ACEL-24-e70002-s002.pdf]
